# Heart Disease

**Published:** 1898-09

**Authors:** 


**Heart Disease.** By Sir WILLIAM H. BROADBENT, Bart., M.D., F.R.S., and JOHN F. H. BROADBENT, M.D. Pp. 331. London: Baillière, Tindall and Cox. 1897.

The portion of this book which deals with prognosis consists of lectures which Sir William Broadbent delivered many years ago. His son has contributed the parts relating to treatment, and has made the rearrangements necessitated by this addition. In a work thus produced by the labours of two physicians at different periods, it is impossible to allot to each the part for which he is responsible. If we deal with it as expressing the views of Sir William, we are bound to say that it is hardly worthy of one who has proved himself a careful observer and an original thinker.

While we read the book with respect as the work of a physician of repute, yet all the statements must not be taken as well-established facts. For example, Sir Wm. Broadbent can hardly have tested his clinical observations in the *post-mortem* room, when he says that dilatation of the left auricle gives increase of dulness to the left, in the third and fourth intercostal spaces. Again, absence of impulse of the right ventricle is spoken of as one of the signs that the left ventricle only is acting, whereas it is doubtful whether the right ventricle is capable of giving rise to an impulse, except in cases of very unusual hypertrophy. Reduplication of the first sound is also very definitely stated to be due to asynchronous action of the two ventricles, without any mention of the fact that physiologists and almost all clinical observers of heart disease believe such an explanation of the reduplicated sound to be impossible.

Of the various other clinical and pathological points which might be controverted we will limit ourselves to one. In discussing the diagnosis of fatty degeneration of the heart, it is stated that after a brisk walk the fatty heart "goes to pieces," and the pulse becomes irregular and shorter than before. It must be obvious that in a failing heart, from any one of various causes, irregularity of the pulse will be brought out, or intensified, by over-exertion. A physician who lays stress upon such a sign cannot be aware of the rarity of the fatty heart in the *post-mortem* room, and must have frequently diagnosed the disease when it did not exist.

We are glad to note, however, that to several interesting points we believe to be true, but are not generally recognised, Sir Wm. Broadbent adds the weight of his authority. Amongst others, he mentions that the presence of murmurs after an attack of rheumatism must not be taken as conclusive evidence of permanent organic disease. He also states that from murmurs alone it is impossible to diagnose aortic stenosis, and further notes the presence of loud basic murmurs over the pulmonary area where anæmia and congenital stenosis of the pulmonary artery can be excluded.

